# Performance of a bioglass-based dentine desensitizer under lactid acid exposition: an in-vitro study

**DOI:** 10.1186/s12903-018-0642-z

**Published:** 2018-11-21

**Authors:** Manz Andrea Stefanie, Attin Thomas, Sener Beatrice, Sahrmann Philipp

**Affiliations:** Clinic for Preventive Dentistry, Periodontology and Cariology, Center of Dental Medicine, University of Zurich, Plattenstr. 11, 8032 Zurich, Switzerland

**Keywords:** Dentine sensitivity, Hypersensitivity, Periodontitis, Desensitizer, Electron microscopy

## Abstract

**Background:**

Dentine hypersensitivity is especially frequent in patients with pronounced periodontal attachment loss. Aim of the treatment is an obstruction of the dentine tubules in order to inhibit liquid or osmotic motion, which is considered as trigger for pain sensations. Novel approaches aim for obstruction by calcium phosphate compounds in order to rely on biocompatible compounds. It was the aim of the study to optically investigate the morphology and to assess the fluid permeability of treated dentine surfaces.

**Methods:**

Dentine discs were pretreated in an ultrasonic bath with 17% EDTA to clean the lumina of the dentine tubules. Samples of group A remained untreated while Seal&Protect® as a conventional desensitizer was applied for group B and DentinoCer in group C. Discs were mounted into a pulp fluid simulator (PFS) with a methylene blue solution in order to create a flow pressure of 0.5 bar. Over 12 d, discs were exposed three times per day to 0.1 M nonsaturated lactic acid. At baseline and after 2, 8 and 12 d samples were removed from PFS and prepared for SEM analysis. Tubule obstruction was assessed quantitatively using Olley scores and by qualitative description of the surface. Absorption spectrometry was used to assess the concentration of leaked methylene blue outside the samples in order to estimate dentine permeability.

**Results:**

Untreated discs showed clean lumina of all tubules at all time points and magnifications. From day 2 onwards dentine showed exposed collagene fibers due to acid exposition. Seal&Protect® initially showed homogenous dentine surface coverage that got a more granulomatous aspect in the course of treatment time. Few samples showed sporadic tubules with open lumen at day 8 and 12. Group C showed samples with a homogeneous, even surface. Narrow slits in the superficial layer are visible from day 4 on, but the dentine surface remained invisible and dentine tubules were closed till the end of the investigation period.

**Conclusion:**

Over 12 d of lactid acid exposure, samples showed complete coverage of the dentine tubules in the chosen in-vitro-model when treated with Seal&Protect® or DentinoCer.

## Background

Dentine hypersensitivity is defined as a short, sharp pain caused by exposition of dentine tubules to thermal, tactile, chemical or osmotic stimuli, and that is not referable to any other pathology [1]. According to the hydrodynamic pain theory liquid fluctuations caused either by rapid changes in temperature or osmolarity activate nerve endings in the pulpal tissue, what is – after centric conduction of the stimulus - experienced as pain [2].

Epidemiologic data for the prevalence of dentine hypersensitivity generally show a broad range between 3 and 98%. The range is a result of different inclusion criteria, assessment protocols and different populations of the regarding studies [3]. In patients that have received periodontal treatment, several predisposing factors result in an increased prevalence: In the first place, gingival recessions due to periodontal attachment loss are a typical finding in patients with periodontitis. They result after attachment loss due to inflammation of the periodontal tissues, when therapy results in detumescence of previously swollen soft tissues. The loss of the gingival collar results in exposure of the cervical tooth area, that previously had been covered by well perfused and therefore thermically isolating tissues. Secondly, repeated iatrogenic instrumentation and intense daily brushing of these areas during the periodontal maintenance therapy causes a progressive loss of dental hard tissue, since the regarding tooth area is not protected by hard-wearing enamel but by rather soft and easy abrading dental cementum and dentine. The combination of these impairments is – of course - aggravating the pain symptomatology [4–6]. In particular, erratic tooth brushing techniques and usage of abrasive toothpaste, repeated exposure to erosive nutrition and lactic acid due to the bacteria-induced decomposition process of short-chain carbohydrates are additionally aggravating the affliction. As long as dentine hypersensitivity comes along without or with only minor dental hard tissue defects, the pain sensations can often be successfully treated by topical application of so-called desensitizers: Usually, varnishes cover the exposed dentine tubules and might also superficially penetrate the porous dentine surface [7]. Then, preparations with a high wetting potential are able to penetrate the dentine tubules. Penetration is facilitated by diffusion of highly concentrated solvents like HEMA [8] or PENTA [9]. In the tubules, the compounds precipitate as solid particles that are supposed to bind to the inner tubule surface. Hence, tubules occlude and the evocation of a pain sensation is hampered due to a disabled liquid fluctuation. Even if modern desensitizers clinically work well directly after application, for most of the products the effect begins to fade already after several days [10, 11]. Furthermore, the effect of products like Gluma® (Haereus Kulzer, Hanau, Germany) or ShieldForce® (Tokuyama Dental, Tokyo, Japan) relies on the precipitation of particles like hydroxyethyl methacrylate (HEMA) or triethylene glycol dimethacrylate (TEGDMA) on one hand and Bisphenol-A-glycidyl-dimethacrylate (BisGMA) on the other hand. These compounds remain embedded in the host tissues. They are, however, considered to be highly biologically effective, what might lead to serious undesired effects: In the case of BisGMA localized cytotoxic effects have been shown in in-vitro models [12]. Even products with comparatively low toxicity like Seal&Protect® (Dentsply GmbH, Konstanz, Germany) bear the risk of allergic reactions to ingredients like methacrylates or photoinitiators like camphorquinone [13]. Furthermore, estrogene-like activity of BisGMA [14] and mutagene activity of TEGDMA [15] have been suspected based on results from in-vitro studies.

For both reasons, the temporary desensitizing effect and the disadvantage of incalculable side effects, more biocompatible and longer lasting mechanisms for desensitizers are needed.

Accordingly new approaches were designed in order to regenerate dentine-like material in the tubules instead of filling them with potentially harmful substances. DentinoCer contains soluble calcium phophate bioglass diluted in a slightly alkaline gel. Due to its basic character, the saturation concentration for calcium and phosphate are kept up. According to the developper’s conception this might render new formation of apatite on the exposed root surface and in the tubules possible.

It was therefore the aim of our study to assess occlusion of dentine tubules treated with DentinoCer in a simple in-vitro-model, that simulates acid exposure as due to bacterial decomposition of short carbohydrates.

## Methods

### Preparation of the specimen

Bovine teeth were harvested from anterior parts of bovine jaws purchased from the slaughterhouse Zurich, Switzerland, where cattle had been slaughtered the same day. Front teeth were extracted from the jaws using dental elevators and cleaned with hand curettes. Extracted teeth were stored in tap water at 5.0 °C until further processing.

Circular dentine discs of a diameter of 3 mm were cut out with a trephine from the cervical area of the teeth. A total of 60 bovine dentine discs were prepared. Discs were then centered in a round silicon form of a diameter of 5 mm and coated by methacrylate, carefully avoiding resin contamination of the dentine surface. The specimens were then grinded to a thickness of 1.5 mm and the surfaces were gradually smoothened with 2′000 grit and 4′000 grit paper (Struers waterproof SiC, Birmensdorf, Switzerland) in a water-cooled grinding wheel (Struers, Tegramin-30).

**Fig. 1:**
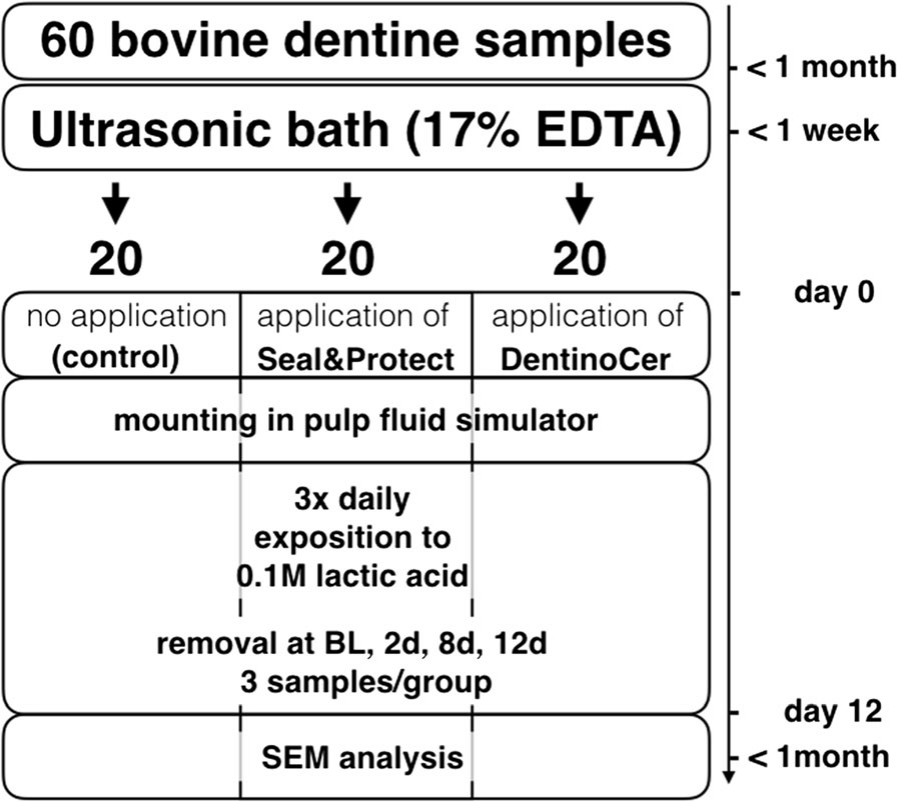
Flow chart of the study design. EDTA – Ethylene diamine tetra acetic acit, BL – baseline, SEM – scanning electron microscope

The specimens were then cleansed in a 17%-EDTA ultrasonic bath for 10 min and afterwards gently brushed with a soft toothbrush (Curaprox Supersoft, Curaden, Dietikon, Switzerland) while rinsing with abundant tab water in order to receive a nearly bacteria-free surface and unobstructed dentine tubules. The dentine samples were then exposed to a gamma radiation of 12 kGy for 34.6 h in order to avoid bacterial overgrowth during the course of the study. Specimens were stored in tab water during preparation in order to avoid exsiccation of dentine before further processing (Fig. 1).

#### Experimental set-up

In order to simulate the pulp fluid pressure of the vital tooth, 60 PVC tubes with a length of 0.6 m and an inner diameter of 7 mm were placed vertically in a custom-made stand and filled with an artificial dentinal fluid (ADF) [16].

PVC tubes had previously been gas-sterilized with ethylene oxide (3 M Schweiz, Rueschlikon, Switzerland) for 24 h, and all ingredients for the ADF were filtrated sterile (Filtropur VSO 0.2, Sarstedt, Nuermbrecht, Germany) in order to minimise bacterial overgrowth during the study. At the lower end, in each PVC tube a flexible tube of 4 cm of length and an inner lumen of 5 mm was inserted, and the lower opening was locked by the insertion of one dentine specimen each. On this behalf, a custom-made applicator allowing for easy and sure disc handling and insertion was used. Disc surfaces were not touched neither by fingers nor forceps, what might have recontaminated or destroyed the disc surfaces. Then, the prepared tube ends with occluding discs were placed into measuring glasses with 150 ml sterile tab water each, in order to keep the discs wet.

In each tube, the fluid level was regulated on a height of 0.5 m, resulting in a fluid pressure of 0.5 bar.

In order to minimise air-born contamination the whole experimental setup was placed under a laboratory hood throughout the investigation. All involved operators wore medical gloves, surgical masks and tied up hair during any manipulation.

#### Pre-treatment of the dentine specimens

Sixty specimens were randomly distributed into three groups of 20 each as follows:

A. (control): No treatment of the dentine surface.
B. (Seal&Protect®): The surface of the specimens was gently dried by oil-free airflow. Then Seal&Protect® (Heraeus Kulzer, Hanau, Germany) was applied with a sterilized brush applicator (Orbibrush, Orbis, Muenster, Germany). The specimens were left untouched with some liquid excess on the surface for another 20 s before the samples were gently dryed by airflow for 5 s. Finally, the surface was light-cured from a distance of 5 mm for 10 s at a wavelength of 380–515 nm and an intensity of 1200 mW/cm2 (bluephase G2, Ivoclar Vivadent AG, Schaan, Lichtenstein). Directly thereafter, a second application was performed accordingly and the discs were cured for another 10 s without any airflow application.
C. (DentinoCer): The specimens’ surface was dried by gentle oil-free airflow. After vigorous shaking of the vial, DentinoCer (Biocer, Bayreuth, Germany) was applied with a sterile brush applicator (Orbibrush, Orbis, Muenster, Germany) for 5 min and left for another 5 min untouched.

The test liquids used for group B and C had previosly been gamma-sterilized by a radiation of 23 kGy.

**Fig. 2:**
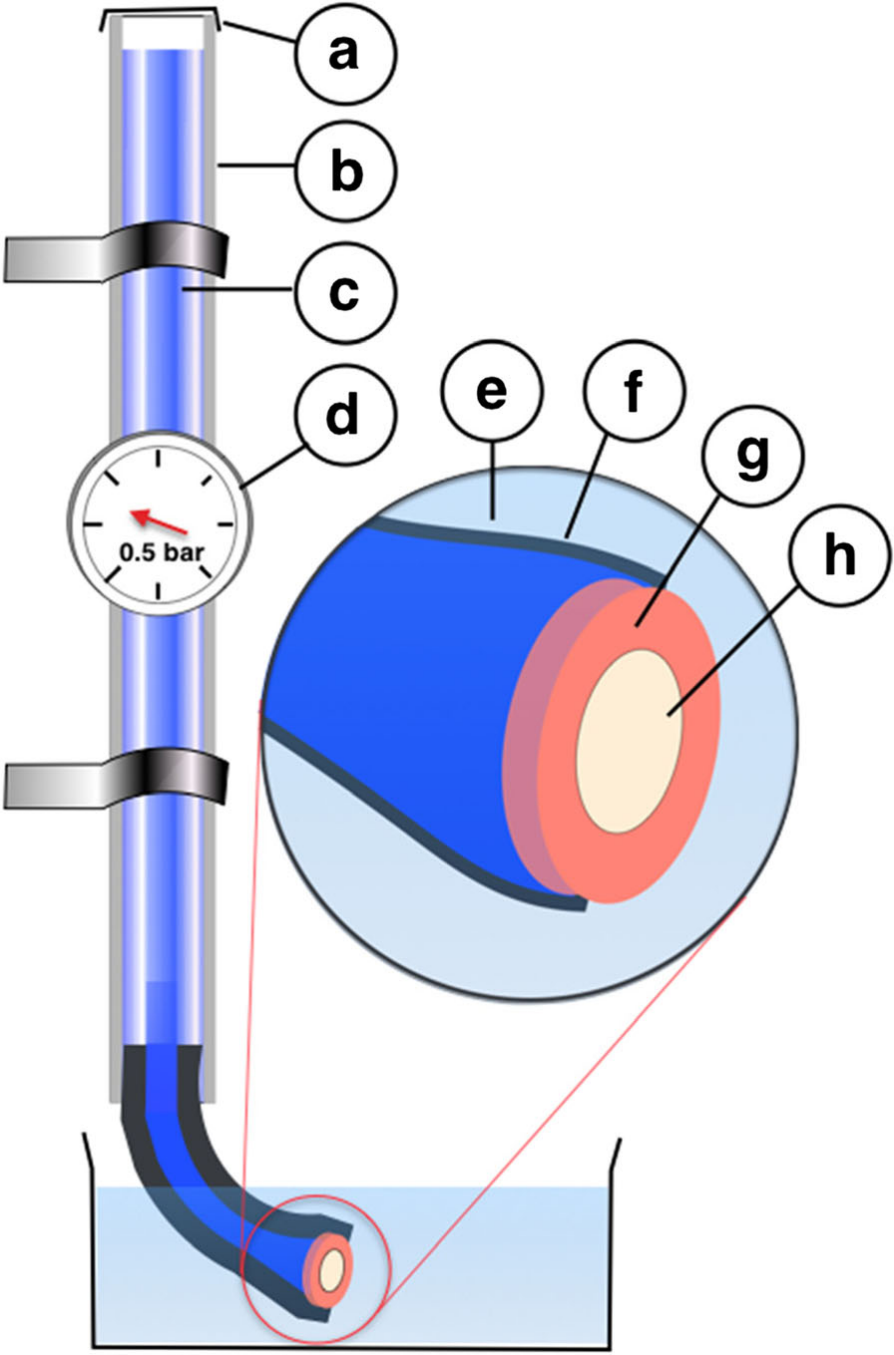
Experimental set-up. **a** – baring; **b** – stiff tube; **c** – artificial saliva; **d** – liquid column pressure set at 0.5 bar; **e** – tap water; **f** – flexible tube; **g** – methacrylate socket; **h** – dentine disc

Immediately after treatment, the samples were placed back into the tube endings and into measuring glasses containing sterile tap water (Fig. 2).

#### Exposition to lactic acid

The tube endings including the specimens were taken out of the tap water and placed into measuring glasses with buffered sterile lactic acid (pH 5) for 10 min for three times a day with a minimal time lag of 5 h. Subsequently, the samples were gently rinsed with sterile tap water and placed back into the measuring glasses. Once a day, liquid samples from the tap water were drawn for spectrometric assessment. The residual water was discharged, the glasses cleaned and refilled with 150 ml of fresh sterile tap water.

Five discs from each group were removed from the set-up immediately after pre-treatment and defined as baseline samples. In the following, further 5 samples each were removed at day 4, 8 and 12.

Once removed from the experiment, samples were stored in 2.5% glutaraldehyde solution until they were further processed for imaging.

#### Imaging

Before imaging, samples were fixated using a 2.5% phosphate-buffered glutaraldehyd solution for 24 h, rinsed 3 times in phosphate buffer solution to remove glutaraldehyd remnants and exsiccated in an ascending ethanol row of 50 to 96%. Over a time period of 72 h the samples were saturated and fixed in one part resin (Technovit 7200 VLC, Haereus Kulzer, Hanau, Germany). Then, the samples were mounted on specimen holders and sputter-coated with gold (Sputter CCU-010, Safematic GmbH, Bad Ragaz, Switzerland) with a programmed layer thickness of 8.0 nm.

Two different surface analyses were performed:

Top view surface analysis was performed at 10 kV (Zeiss Supra 50 VP, Zeiss, Oberkochen, Germany). Images of the disc surfaces were prepared at a magnification of 5000x and 20,000x. Primary outcome parameter was the assessment of dentine tubules occlusion as proposed by Olley et al. [17].

In order to specially assess the samples by vertical intersection at baseline and after 12 d, samples, which had already been gold-sputtered for the top view analysis, were centrically cut with a diamond saw (Buehler, ISOMET® low speed saw, Prüfmaschinen AG, Dietikon, Diamant Cut-off Wheel, Struers GmbH, Birmensdorf, Switzerland). The samples were embedded in resin and the cut face was grinded and polished with 3000 grit paper as already described. Samples were then vapour-coated with coal particles in order to allow for enhanced discrimination by backscatter analysis, before the intersections were analyzed at 10 kV (Zeiss Supra 50 VP) and images of a magnification of 1000x and 2000x were made.

#### Surface analysis

Top view and intersectional surface morphology and texture were characterized descriptively. For the top view, the classification of Olley was used in order to assess the grade of tubule occlusion.

#### Spectrometric assessment

On each of the 12 study days, 20 ml of the tap water, in which the disc-locked ends of the tubes were placed, were extracted, labelled according to the extraction time before the rest of the tap water was discharged and replaced by fresh water.

Sampling water from same groups and same time points were pooled and concentrations of methylene blue, that had stained the tapwater blue due to tubular leakage through the dentine discs, were assessed by spectroscopy. In a double-beam spectrophotometer (I2010, Portmann Instruments, Biel-Benken, Switzerland) the translucency absorption at 664 nm was assessed. Each measurement was performed threefold and averaged for each group and point of time.

#### Statistics

To compare the data from the spectrometric analysis, Mann-Whitney U test for unpaired tests of not parametrically distributed data was used to check for inter-group differences (i.e. different groups at same days). For these assessments, the level of significance was set at 5%.

## Results

### Surface analysis

#### Group A (control)

**Fig. 3:**
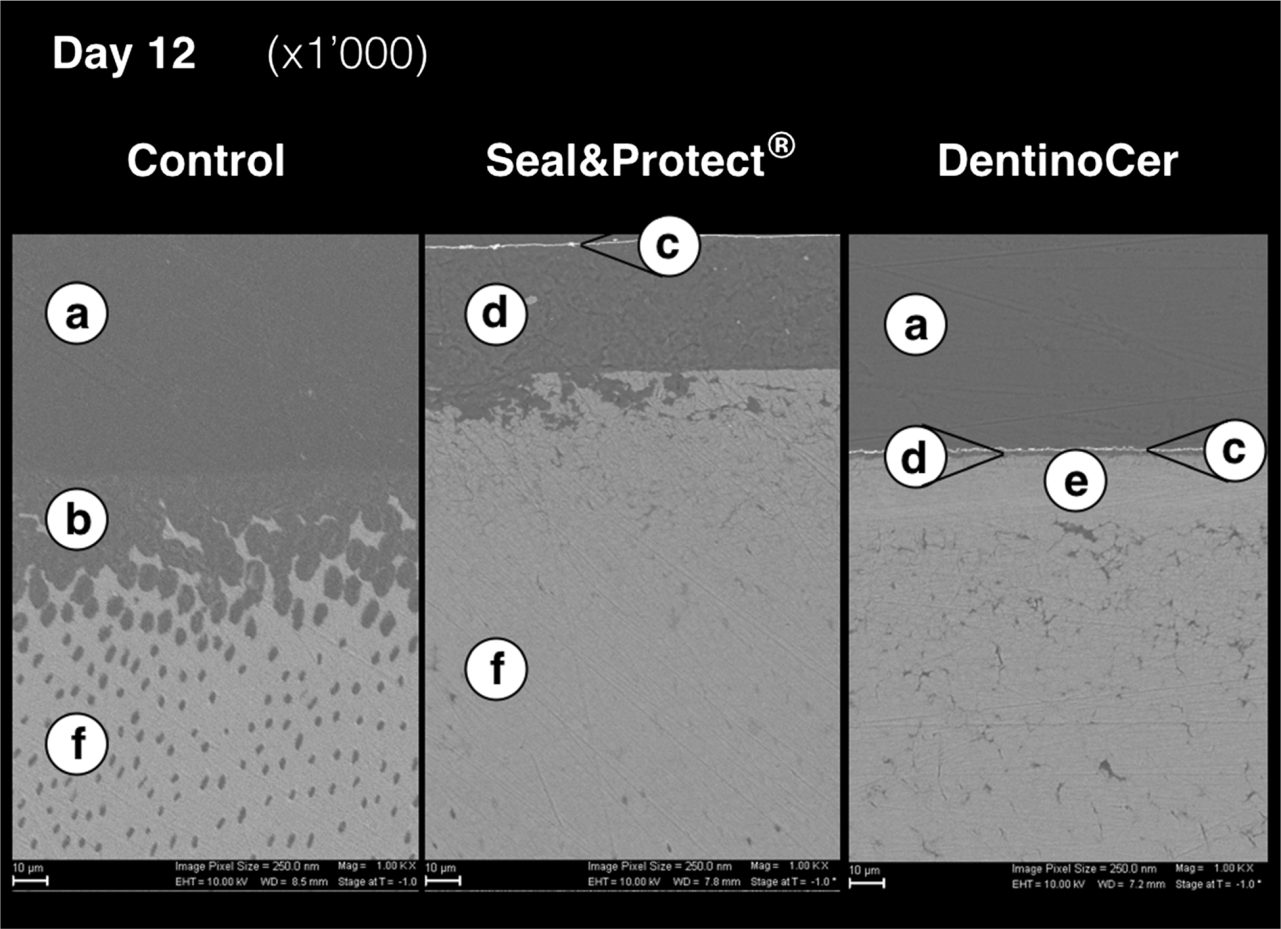
Sectional view of the vertically cut samples after 12 d at 1000-fold magnification. **a** – Technovit imbedding; **b** – Diagonally cut, previously etched dentine tubules; **c** – gold layer deriving from previously performed horizontal sputtering; **d** –layer of previously applied desensitizers; **e** – dense dentine layer without visible dentine tubules; **f** – deep portion of dentine

Top view surface analysis of the baseline samples revealed open tubules, which were clearly visible at 10′000x magnification (Fig. 3). The tubules displayed free and clean lumina in all images. Likewise, the samples showed open tubules after day 4, 8 and 12. The pictures at a magnification of 20′000 show denudated collagen fibers as an effect of the exposure to lactic acid. From day 4 on some rod-shaped bacteria are visible on the dentine surface. Since all the dentine tubules were perfectly open the samples for all points of time depict an Olley score 5 (Fig. 5 and Table 1).

**Table 1:** Top view occlusion assessment of dentine tubules

Group	baseline	4d equivalent	8d equivalent	12d equivalent
Controls	5	5	5	5
Seal&Protect	1 (2)	1 (2)	1 (2)	1 (2)
DentinCer	1 (2)	1 (2)	1 (2)	1 (2)

Olley scores: 1 – occluded, 2 – partially unoccluded, 3 – equally occluded/unoccluded, 4 – partially occluded, 5 – unoccluded [17]. Values in brackets were found very sporadically

**Fig. 4:**
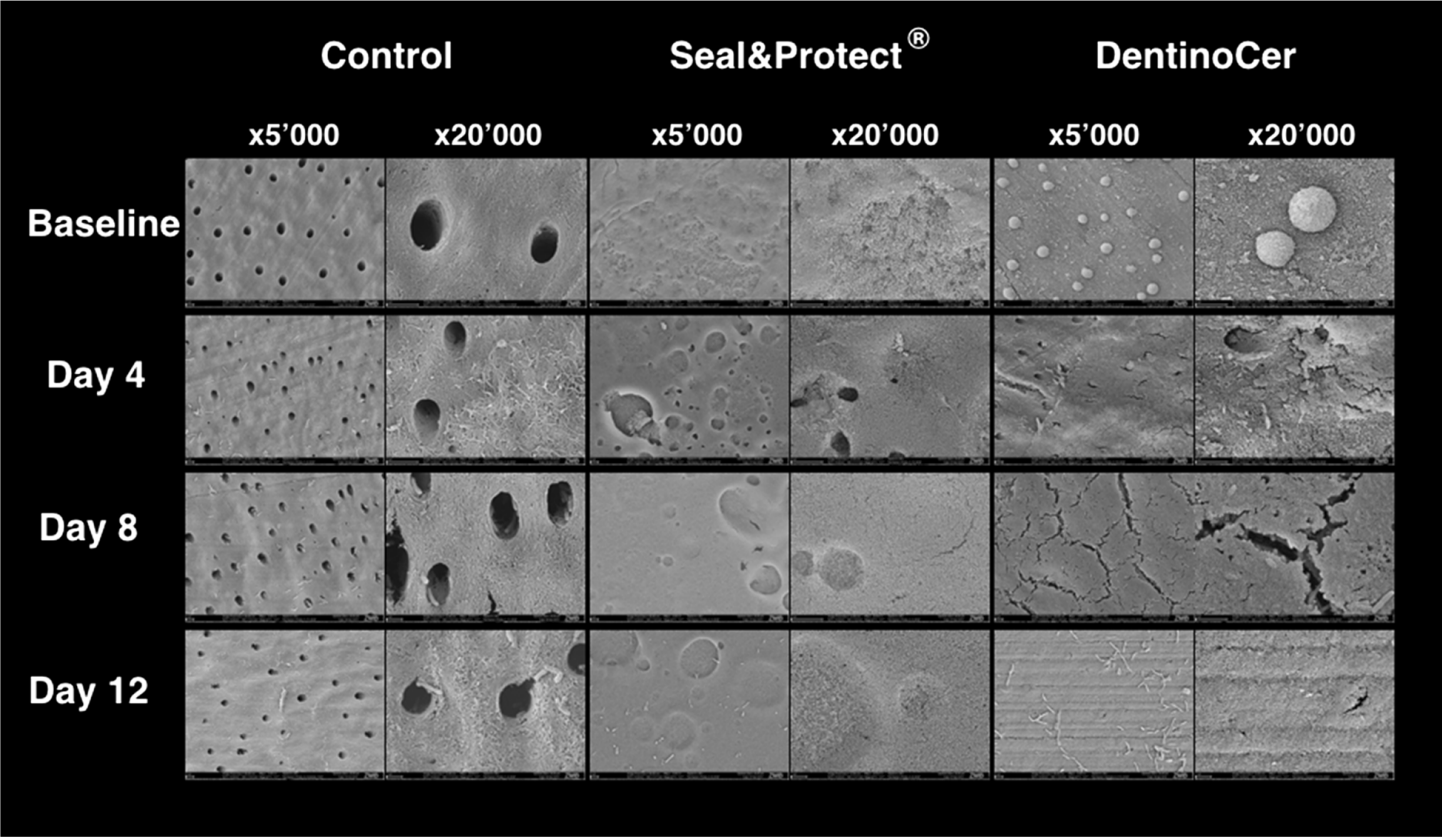
Top views of the samples at different time points. SEM-pictures of top viewed samples of group A (control), B (Seal&Protect®) and C (DentinoCer) at baseline, Day 4, Day 8 and day 12 (D) at different magnifications (× 5000 and × 20’000)

Vertically grinded sample sections after 12 d of repeated lactic acid attack show light grey dentine areas and cut tubules (Fig. 4). Lighter inner tubule walls appear next to dark grey tubule lumina, identically to the area beyond the dentine surface, depicting bubble-free Technovit. On some samples, in a distance of about 2–10 μm beyond the dentine surface drying cracks appear as artifacts due to sample manufacturing. Sporadically, cracks enter the dentine. Generally, the tubule endings at the dentine surface are confluent and show wide-open deltas (Fig. 4), depicting the demineralized area due to an overall of 36 times of acid attack.

**Fig. 5:**
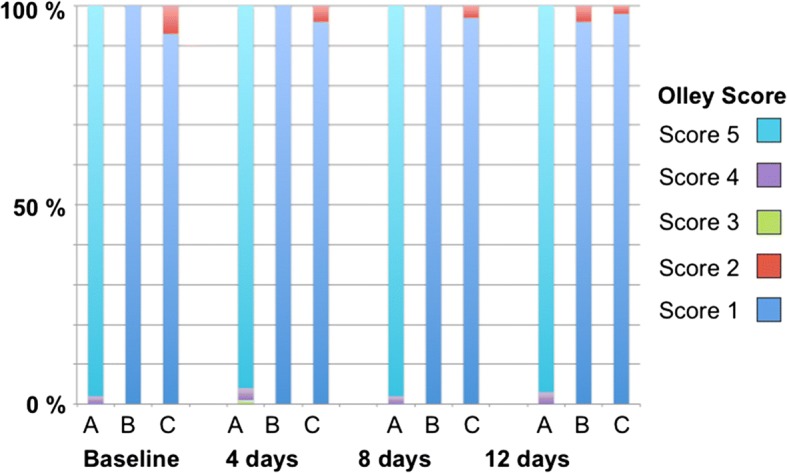
Percentages of Olley scores of the differently treated groups at different times. Olley scores: 1 – occluded, 2 – partially unoccluded, 3 – equally occluded/unoccluded, 4 – partially occluded, 5 – unoccluded [17]

#### Group B (Seal&Protect®)

At baseline, samples show a quite homogeneous smooth surface at low magnification. On some samples, very few larger (ca. 2.0 μm) and deep pores equal in size with the apertures of the dentine tubules with the untreated samples are visible (Fig. 3). During the exposition to lactic acid, the smooth surface shows a more and more granular appearance. On day 8 and 12 on some samples rod-shaped bacteria appear on the surface (Fig. 3). Over the whole treatment time, the Olley score of the dentine surface with fully covered tubules (with very few exceptions on one sample) in all the images is 1 (Fig. 5 and Table 1).

SEM-images of vertically ground samples from day 12 show a brighter, cloudy greyish coverage next to the dentine surface, indicating a compact layer of Seal&Protect of at least 50 μm (Fig. 4). This layer is covered by a thin optically dense line that indicates the gold sputtered surface of the vertically cut sample (Fig. 4). Though the openings of the dentine tubules show the same greyish filling, it is not clear whether deeper parts of the tubules are filled with the more homogeneous Technovit or are infiltrated by the test substance.

#### Group C (DentinoCer)

The top view pictures of the samples treated with DentinoCer initially show a homogeneous, flat surface at baseline without visible tubule openings at low magnification. Spherical particles of a size of 2–3 μm appear on the surface. At 20′000x magnification, some inhomogeneous slits of a length of 1–3 μm become visible (Fig. 3). Though these slits become more frequent at day 4 and 8 at higher magnification, the surface seems generally to be completely covered (Fig. 3). Stripe patterns are also visible on several samples. No open tubules and no exposed dentine surface are visible. After 8 and 12 d rod-formed bacteria appear on the otherwise unchanged surface. After 12 d bacterial colonization is more frequent and at 20′000x magnification the surface seems rough now, exposing a cotton wool-like pattern, in its appearance clearly different from the naked dentine surface of group A, with bacteria that have entered the superficial layer (Fig. 3). Since no tubule openings are visible, the Olley scores range between 1 and 2 (Fig. 5 and Table 1).

Vertical images show a constant, about 2–3 μm thick layer over the dentine surface depicting the glass matrix of the applied solution (DentinoCer, Fig. 4). A very thin, optically dense (white) line shows the gold sputtering of the treated dentine surface (d). Beyond this line a homogeneous line represents the Technovit embedding of the sample (a). Most samples revealed a 20–100 μm thick dentine layer with an inhomogeneous dentine-like cloudy appearance that do not show any tubules (e). Deeper, beyond that dense layer, dark dentine tubules are again visible throughout the deeper dentine sample (Fig. 4).

#### Spectrometric assessment

**Fig. 6:**
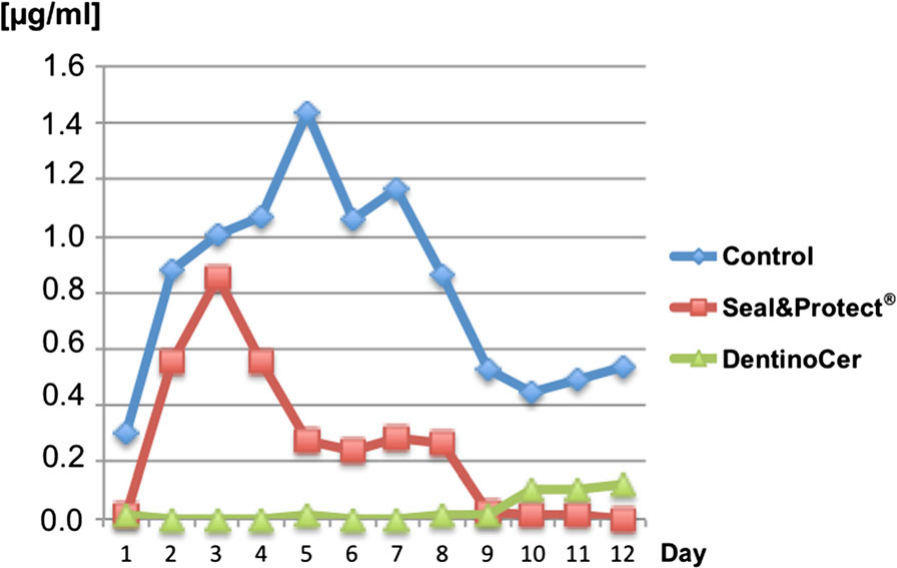
Concentration of leaked methylene blue. Mean concentrations of leaked methylene blue [μg/ml] of the three groups recorded at each day. Since the three spectrometric measurements of each group/point of time showed only minimal deviations, no standard deviations are indicated. Intragoup values of different points of time are connected by a line for better illustration

Assessment of methylene blue from the measuring glasses show high initial mean values of 0.3 μg/ml for color penetration for group A (control), that rises to a maximum of 1400 μg/ml at day 5 before decreasing again. For group B *(*Seal&Protect®), the concentration of methylene blue rises from 0 to 0.8 μg/ml on day 3, and decreases to 0 during the residual investigation period. Color penetration for group C (DentinoCer) was generally very low and reaches a late maximum of 0.1 g/ml from day 8 on Fig. 6. At the first days the penetration values for the test groups reached statistically significant differences (*p*-values of 0.046 at day 1 and 2) and then again from day 9 on (p-values 0.046 at day 9, 10 and 12, 0.043 at day 11).

## Discussion

The present study is the first to assess the effect of a topical application of a novel dentine desensitizer based of a regenerative approach using a bioglass matrix. Seal&Protect® was taken as comparing solution because of its resistance and its resistivity [18, 19]. Products were applied on bovine dentine samples that, connected to a pulp fluid pressure simulator, were periodically exposed to lactic acid. Results show, that a homogenous layer covered the openings of previously free tubules after one application only. The tubules remained closed after repeated exposure to lactic acid over a study period of 12 d, corresponding to a total exposition time of 6 h. The assessment of specimens that had been cut in vertical direction, i.e. parallel to the direction of the tubules, showed the apposition of a superficial matrix layer of 2–3 μm on the dentine surface. Furthermore a 20–100 μm thick layer emerges within the dentine, where tubules were not optically detectable, thus suggesting their obturation with a radio-opacity similar to that of dentine. Thereby, the assessment of the effect of the treatment on the outer dentine surface was combined by an assessment of deeper dentine layers to a depth of around 50–100 μm. Though the finding, that dentine seems to show superficially obturated tubules, optical analysis may not answer the question whether obturating material is the matrix of DentinoCer or if newly formed calcium phosphate compounds have precipitated.

Likewise, it is discussable if not unfavourable orientation of the tubules might render them invisible in the reported greyish zone. Regarding this issue it is important to state, that this greyish area was not observed in deeper dentine areas, eventually leaving space for a belt with visible tubules more marginally in the dentine. Neither was the reported layer observed in one of the samples of group A (control) or B *(*Seal&Protect®).

In order to further assess the physical permeability, the passage of methylene blue was assessed in the study set-up, simulating pulp fluid pressure based on a previously published model bei Jungbluth et al. [16].

Even though the study design was adapted as best possible to the clinical situation, several issues depict compromises and have to be discussed: Previous to the application of dentine desensitizers, dentine discs had been pretreated in order to open and clean the dentine tubules. Figure 1 shows that cleansing with EDTA resulted in perfect purification of the tubule lumina. Thus however, samples at baseline depicted a situation that is much worse in terms of osmotic and fluid movements as compared to the clinical situation, where tubules are at least partially filled with organic compounds, toothpaste particles and dentine detritus due to brushing [20]. Therefore, this first study to assess the application of DentinoCer started from a more difficult baseline situation as compared to the clinical situation. This fact renders the observed complete tubules closure over several days clinically even more promising with regard to the possible desensitizing effect.

Since pre-study data showed substantial problems due to bacterial overgrowth after already few experimental days, thorough care was taken to decontaminate specimens, desensitizers and the whole tube system by which the pulp chamber pressure was simulated: The dentine discs had been exposed to a radiation of 12 kGy, while tubes, instruments and applicators which were used during the experiment had previously been gas-sterilized. Applied liquids, Seal&Protect® and DentinoCer were gamma-sterilized by a total of 23 kGy. The liquid in the tubes, which simulated the pulp fluid pressure, contained sterile water and only ultrafiltrated components. Nevertheless, first bacteria were detected on day 4 and – in higher numbers – on day 8 and 12. Especially group C, which was treated with the experimental liquid DentinoCer, showed bacterial colonization. Though the exact origin of these bacteria is not clear, airborne bacterial contamination or contamination directly by manipulation during exposition to acid is possible. Since the coloured fluid which simulated the pulp chamber pressure is one of the possible sources, different effects on the study set-up are possible: On the one hand, bacterial overgrowth in the tubes might have led to lower or even suspended pulpal fluid. On the other hand it is possible that due to bacterial growth in the surface layers the initial sealing due to application of the test liquids got hampered or that present obturation degraded due to bacterial metabolism, colonization and movement. In any case, the observation that finally only few bacteria were detected suggests a rather small effect of those microorganisms for the present investigation. If the source of bacteria was DentinoCer itself, what might still be possible despite very strong exposure to radiation [21], a clinical effect in the oral cavity with a plethora of quickly proliferating and highly adapted bacterial species does not seem to constitute a matter of great concern – as long as no specific pathogens are harboured in the liquid [22].

Like in every study with an optical assessment of individual samples, some inhomogeneity for SEM-imaging was found within each group. Accordingly, in the set of three samples for every group and every time point some areas showed surface morphologies, that seemed “anormal” with regard to the vastly predominating characteristics in the major part of the analyzed surface. Therefore, we found lumina of single tubules in the control group, which still harboured small particles of detritus, or a small area on a sample from group B at day 4 that had not been completely covered by Seal&Protect®. Anyhow, best efforts were taken to generally describe the vastly dominating and therefor “normal” aspects.

Furthermore, cracks were found after 12 d on several samples of the control group. Since these cracks are not filled with Technovit, they must have emerged late in the fixation process, maybe during SEM analysis due to the specific atmospheric conditions in the vacuum chamber of the microscope.

The results from the optical assessment were qualitatively supported by the findings from the spectrometric assessment: While methylene blue penetrated the untreated dentine discs and stained the water in the measure glasses in higher concentrations, staining was low for the test groups and especially for treatment with DentinoCer. Principally, the fact that the application of desensitizing agents allows for residual dentine permeability is in accordance with the respective literature [23, 24]. The reason for the fact that the penetration maximum is at day 2–4 for the untreated discs and the discs of group B before concentrations decline to zero after day 8–12, is unknown. One reasonable explanation is a possible agglutination of the coloured medium in the tubes or in the tubules of the discs. Since in the electron microscope images no such plugs were detectable only a partial obturation of the proximal tubule areas (i.e. distant from the treated surface) beginning after day 5 is possible. On the other hand this would not explain the rising stain penetration till day 5.

The present study aimed to assess the effects of lactic acid on the treated dentine surface in order to simulate cariogenic activity, but no mechanic force was applied to simulate brushing. Since it seems likely that mechanical ablation completely removes the silicate matrix and also have an impact of obturated tubules, future studies should include repeated brushing in combination with lactic acid attack in order to show whether dentine tubules remain closed or if superficially obturating remnants will be washed out, before clinical trials test applicability and pain-reducing effect of DentinCer. Likewise, future studies should assess whether the tubules are filled by the glass matrix itself, or whether a desirable regeneration in terms of a precipitation of calciumphosphate compounds took place.

## Conclusions

Over the 12 d of lactid acid exposure, dentine samples that have been treated with Seal&Protect or DentinoCer showed a complete coverage of the dentine surface. Dentine specimens treated with DentinoCer showed also low tubular leakage during this time. Future studies should assess dentine samples exposed to mechanical stress by brushing and analyze the chemical composition of the obturing material in the lumina of the dentine tubules.

## Abbreviations

ADF: Artificial dentinal fluid
BisGMA: Bisphenolglycidyldimethacrylate
EDTA: Ethylenediamine tetraacetic acid
HEMA: Hydroxyethylmethacrylate
PENTA: Dipentaerythritol pentaacrylate
PVC: Polyvinyl chloride
SEM: Scanning electron microscop
TEGDMA: Triethylene glycol dimethacrylate
