# Correction to: Performance of a bioglass-based dentine desensitizer under lactid acid exposition: an in-vitro study

**DOI:** 10.1186/s12903-019-0855-9

**Published:** 2019-07-24

**Authors:** Andrea Stefanie Manz, Thomas Attin, Beatrice Sener, Philipp Sahrmann

**Affiliations:** Clinic for Preventive Dentistry, Periodontology and Cariology, Center of Dental Medicine, University of Zurich, Plattenstr. 11, 8032 Zurich, Switzerland

**Correction to: BMC Oral Health**

**https://doi.org/10.1186/s12903-018-0642-z**

Following publication of the original article [1], the authors reported their family and given names have been mismatched. The correct names can be found below:

Given name: Andrea Stefanie

Family Name: Manz

Given name: Thomas

Family Name: Attin

Given name: Beatrice

Family Name: Sener

Given name: Philipp

Family Name: Sahrmann

The publisher apologizes for any inconvenience caused by this error.

## References

[1] Stefanie (2018). Performance of a bioglass-based dentine desensitizer under lactid acid exposition: an in-vitro study. BMC Oral Health.

